# Insights into the Evolving Roles of Circular RNAs in Cancer

**DOI:** 10.3390/cancers13164180

**Published:** 2021-08-20

**Authors:** Katherine Louise Harper, Timothy James Mottram, Adrian Whitehouse

**Affiliations:** Astbury Centre for Structural Molecular Biology, School of Molecular and Cellular Biology, Faculty of Biological Sciences, University of Leeds, Leeds LS2 9JT, UK; bs14klh@leeds.ac.uk (K.L.H.); t.mottram@leeds.ac.uk (T.J.M.)

**Keywords:** circRNA, ceRNAs, molecular biology of cancer, miRNAs, fusion circRNA, exosomal circRNA, biomarkers, circRNA therapeutics

## Abstract

**Simple Summary:**

The central dogma of RNA biology was traditionally governed by the transcription of DNA into messenger RNA (mRNA) followed by translation into protein. As additional functions of RNA were discovered, it became clear that there were many species of RNA, and many of those species were multifunctional with roles outside of coding for protein. One such area of discovery was the ability to form circular RNAs (circRNAs) that have been shown to be important in a wide range of cellular processes and as such are important in health and disease. These functions broadly include the modulation of gene expression, facilitation of protein–protein interactions and protein translation. It is important to understand how the dysregulation of these processes are involved in cancer progression and the avenues this may open for the targeting or overexpression of circRNAs as novel therapeutics.

**Abstract:**

The majority of RNAs transcribed from the human genome have no coding capacity and are termed non-coding RNAs (ncRNAs). It is now widely accepted that ncRNAs play key roles in cell regulation and disease. Circular RNAs (circRNAs) are a form of ncRNA, characterised by a closed loop structure with roles as competing endogenous RNAs (ceRNAs), protein interactors and transcriptional regulators. Functioning as key cellular regulators, dysregulated circRNAs have a significant impact on disease progression, particularly in cancer. Evidence is emerging of specific circRNAs having oncogenic or tumour suppressive properties. The multifaceted nature of circRNA function may additionally have merit as a novel therapeutic target, either in treatment or as a novel biomarker, due to their cell-and disease-state specific expression and long-term stability. This review aims to summarise current findings on how circRNAs are dysregulated in cancer, the effects this has on disease progression, and how circRNAs may be targeted or utilised as future potential therapeutic options.

## 1. Introduction

Cancer affects approximately 5% of the population in Western countries and, despite an increasing five-year relative survival rate in the last decade, still accounts for ~14.6% of all human deaths [1]. Understanding the molecular biology underlying the mechanistic causes of cancer is an area of intense research due to the impact cancer has on society and the sheer complexity of cancer biology. Cancer is often described as the transformation of normal cells to malignancy, which requires sequential mutational damage to the cellular genome, resulting in the downstream activation of oncogenes or inactivation of tumour suppressor genes [2]. Furthermore, aberrations in epigenetic processes have been shown to be involved in the initiation and progression of cancer through altered gene function of oncogenes [3,4]. Epigenetic processes that cancer can reprogram include histone modifications, DNA methylation, RNA modification and non-coding RNAs. The broad nature of this disease results in the need for understanding numerous different signalling pathways, gene expression profiles and protein–protein interactions. Decades of cancer research has led to a deep understanding of the molecular mechanisms behind a large number of cancer-related processes. However, to date, research pertaining to the role of noncoding RNAs (ncRNAs) in cancer is less established. NcRNAs, comprise the majority of the RNA transcribed from the genome, and their role in cancer is far less elucidated than coding transcripts and their subsequent proteins. Nevertheless, it is now recognised that ncRNAs perform major regulatory roles within the cell and are widely implicated in cancer biology.

Circular RNAs (circRNAs) are a subset of ncRNAs, characterised by their closed loop structure, that have recently been identified as important factors in the development of various diseases. The abundant and dynamic nature of circRNA expression has highlighted the capability of circRNAs to be involved in many molecular processes. In particular, circRNAs have been shown to be aberrantly expressed in a multitude of different cancers and furthermore can be involved in the regulation of tumourgenesis through RNA sponging, protein interaction scaffolding, mRNA transcription regulation and protein translation [5]. Furthermore, circRNAs are often expressed in a tissue- or condition-specific manner, with these properties correlating with their importance in cancer [6]. This review aims to summarise current research on circRNAs and their multifunctional roles in the molecular biology of cancer.

## 2. circRNA Biogenesis

**Figure 1:**
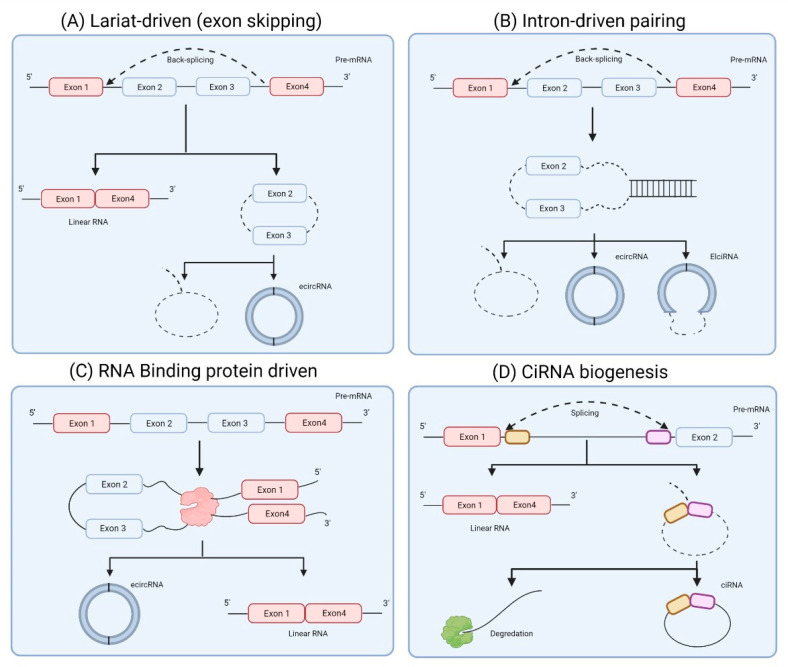
Models of circRNA biogenesis. (**A**). Lariat driven formation. Backsplicing occurs forming a lariat containing introns and skipped exons. The introns can later be spliced out leaving an ecircRNA. (**B**). Intron-pairing driven biogenesis. Complementary flanking introns lead to base pairing bringing splice sites in close proximity promoting circularisation. (**C**). RBP driven circularisation. RBPs through binding to the linear transcript bring splice sites in closer proximity facilitating further factors in promoting circularisation. (**D**). Intronic circRNA biogenesis. circRNA formation is promoted by a range of cis and trans factors leading to formation of an RNA lariat, further processing can remove the 3′ tail forming ciRNAs.

circRNAs are structurally defined by a characteristic covalently closed loop structure that is primarily derived through the backsplicing of canonical splice sites [7]. Flanking inverted repeats, Trans-acting RNA binding proteins (RBPs) and long flanking introns act as backsplice initiation sites (Figure 1). Backsplicing functions through joining the 3′ splice donor site to the upstream 5′ splice acceptor site resulting in a closed circRNA transcript. The process of backsplicing is generally coupled with canonical splicing, as many circRNAs are formed from internal exons of pre-mRNAs. The generation of circRNAs can arise from exons or introns through a number of different pathways. Mammalian exonic circRNA formation has two distinct mechanisms, “direct backsplicing” (Intron-pairing) or “exon skipping” (Lariat driven) [8]. Direct backsplicing is considered the primary mechanism of exonic circRNA generation and involves the standard splicing of two introns, followed by the base pairing of flanking intronic inverted repeats. Subsequently, the 5′ intron branch point attacks the splice donor within the 3′ intron. The 3′ intron in turn repeats this process, attacking the 5′ splice acceptor, forming the circRNA. Exon skipping involves the alternative splicing of exons resulting in the formation of a lariat. The lariat intermediate RNA is often degraded. However, in some cases, it can be further spliced, removing the introns, increasing the proximity of inverted repeat backsplice sites, allowing circularisation and thus forming an exonic circRNA [9].

Intronic circRNA genesis follows a broadly similar mechanism, with circRNA formation promoted through proximal intron splice sites, forming a lariat intron. Subsequent evasion of debranching and degradation allows the 3′ tail of the RNA lariat to be removed increasing stability of the circRNA [10].

## 3. Competitive Endogenous RNAs and Cancer

circRNAs have the potential to act as key competitive endogenous RNAs (ceRNAs) of miRNAs and other small regulator RNAs, and as such may be crucial regulators in cancer. Research has previously focused on the direct interactions between miRNAs and target mRNA dysregulation in cancer, however, the interactions between ncRNAs regulating these processes further upstream is still relatively unexplored.

**Figure 2:**
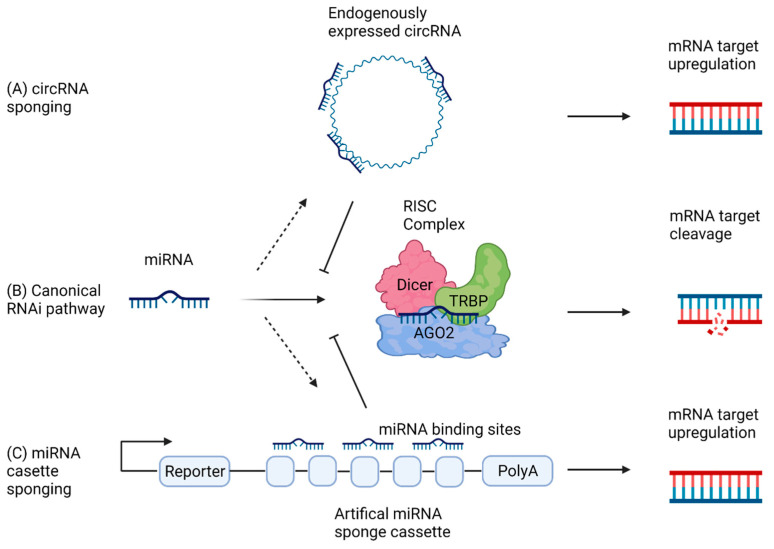
Model of miRNA sponging pathways. (**A**) circRNA sponging occurs upon expression of circRNA containing multiple miRNA target regions that function to sequester miRNA resulting in mRNA target upregulation. (**B**) The canonical RNAi pathway sees miRNA being loaded into the RISC complex allowing targeted degradation of corresponding mRNA resulting in down regulation. (**C**) miRNA sponging can be induced artificially through the expression of a miRNA cassette that contains multiple miRNA target sites for the miRNA of interest. Reporter genes are often included for selection or screening.

Small regulatory RNAs are a class of RNAs that include endogenous microRNAs (miRNAs), small interfering RNAs (siRNAs) and PIWI-interacting RNAs (piRNAs). miRNA biogenesis involves the cleavage of imperfect ~33 nt RNA hairpins into pre-miRNA stem-loops by Drosha and associated co-factors forming the Microprocessor complex (Figure 2) [11]. Alternatively, splicing of pri-miRNA transcripts can form mirtrons that mimic pre-miRNAs and allow downstream processing without Microprocessor complex cleavage [12]. Nuclear pre-miRNAs are exported and further processed in the cytoplasm by Dicer resulting in a mature miRNA/miRNA duplex. One strand of the miRNA duplex associates with a miRISC complex allowing downstream posttranscriptional repression [13]. miRNAs can function as both activators and suppressors of cancer. For instance, miR-15a and miR-16-1 have associations as tumour suppressor genes through the targeting of oncogenic mRNAs in chronic lymphocytic leukaemia (CLL), as the loss of these miRNAs is observed in ~70% of CLLs [14]. While miRNAs expressed from miR-17-92 cluster are classified as oncomiRs, due to their overexpression in a range of cancers, linked to the suppression of tumour suppressor genes [15].

The regulation of miRNA activity can occur via the binding of miRNAs to a miRNA “sponge”. This sponging activity has been shown to be an essential function of some circRNAs [16]. In order to act as a miRNA sponge, the circRNA contains repeating seed-targets complementary to the miRNA sequence. The miRNA binds to the seed target within the circRNA, preventing binding to the canonical mRNA target, instead being sequestered and ultimately degraded [17]. The reduction of miRNA activity on downstream targets can affect cancer pathogenesis through the regulation of signalling pathways or by directly regulating oncogenes or tumour suppressor genes. For example, miRNA sponging activity of the circRNA ciRS-7 against miR-7 results in suppressed miR-7 activity and increased levels of miR-7 targets [18]. ciRS-7 was the first circRNA identified as a ceRNA and has over 70 binding sites for miR-7. Accordingly, increased levels of ciRS-7 in cancer can lead to efficient inhibition of miR-7 functionality. This in turn leads to increased expression of miR-7 target oncogenes Focal adhesion kinase (*FAK*), Insulin Like Growth Factor 1 Receptor (*IGF1R*), Epidermal growth factor receptor (*EGFR*) and Yin Yang 1 (*YY1*) resulting in increased proliferation, EMT and migration. Upregulation of ciRS-7 has been found in a range of cancers including gastric, lung and colorectal [19]. Additionally, within hepatoblastoma, circ-STAT3 increases STAT3 expression through the sponging of miR-29a/b/c/-3p, with this increase contributing to liver malignancy. Interestingly, through its role as a ceRNA, circSTAT3 regulates levels of its parental linear transcript, although most circRNAs do not regulate their parental transcript [20]. There have been over 128 circRNA-miRNA sponging interactions identified that dysregulate oncogenes and tumour suppressor genes, illustrating the miRNA sponging ability of circRNAs can have a profound impact on cell based molecular biology, resulting in cancer pathogenesis.

## 4. Transcriptional Regulation and Cancer

Transcriptional regulation of circRNA begins with the biogenesis of circRNAs and its fundamental co-transcriptional nature. There is a negative correlation between linear and circRNA expression when the majority of exons are circularised, suggesting competition between the linear and the circular transcripts for the spliceosomal machinery [21]. Typically, circRNA abundance is low, however hundreds of circRNAs identified within mouse and human CNS have been shown to be expressed several folds higher than their linear isoform [22]. circRNAs can directly regulate transcription of the linear isoform through variable splicing, involving the preferential splicing of alternative splice sites utilising different splicing methods and mRNA isomers. For example, the co-transcriptional production of circMBL competes with its own Muscleblind (*MBL*) pre-mRNA for the spliceosomal machinery. Conserved MBL protein binding sites are found on the flanking intronic sequences of circMBL. Therefore MBL can stimulate circRNA biogenesis through binding and bridging of flanking introns containing *MBL* binding sites- including its own circMBL and decreasing mRNA levels [21]. Furthermore, the interaction between RNA-binding proteins (RBP) and circRNAs can impact splicing due to the tight regulation of RBPs bound near splice sites. During human epithelial–mesenchymal transition (EMT), circRNA production is dynamically regulated by the alternative splicing factor Quaking (QKI). QKI functions similarly to MBL through the binding of QKI binding motifs flanking circRNA-forming exons that promote circRNA formation in transcripts that are predominantly linearly spliced [23].

**Figure 3:**
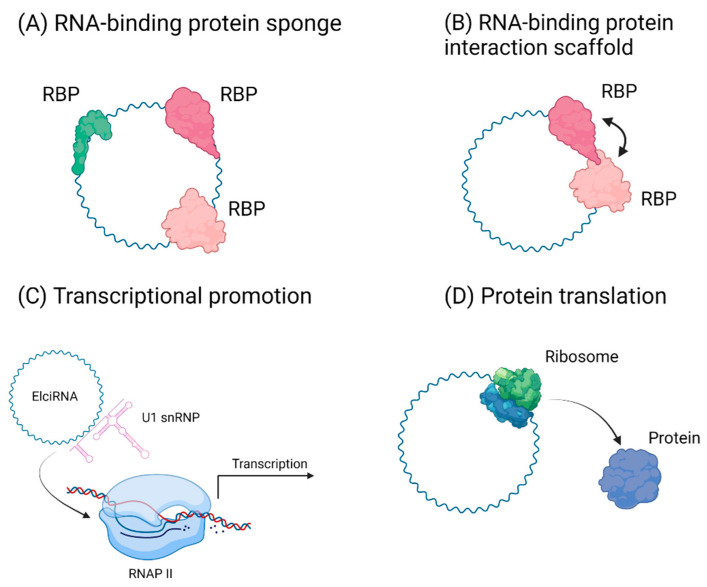
Proposed functions of circRNAs. (**A**) circRNAs can facilitate protein-protein interactions through direct binding. Either acting as sponges preventing normal protein function or (**B**) as scaffolds in aiding protein-protein interactions. (**C**) circRNAs can regulate transcription through recruitment of transcription factors including RNA pol II. (**D**). circRNAs can be potentially translated.

An emerging area of study is the interaction between circRNAs and RNA binding proteins (Figure 3). These interactions have been shown to control gene expression. Human antigen R (HuR), a protein involved in cellular proliferation and development is regulated by circAGO2 through the recruitment of HuR to the 3′-UTR of target genes resulting in downstream proliferation and pathogenesis in gastric cancer cells [24]. Intron-containing circRNAs (EIciRNAs) promote the transcription of parental genes, which is believed to occur via holding RNA–RNA interactions between U1 snRNA and EIciRNA, enhancing interactions with Poll II transcription complex, thereby promoting parental gene expression [25].

A further mechanism of circRNA regulation of transcription occurs via induction of DNA hypomethylation in the promoter region of the parental gene or by regulating intronic enhancers. The regulation of the *FLI1* gene in the development of breast cancer occurs via the FECR1 circular RNA interacting with the *FLI1* promoter through extensive demethylation, promoting metastasis [26].

## 5. circRNAs and Translation

Although classified as ncRNAs, there is evidence that certain circRNAs are able to be translated under certain conditions, although this is still controversial. circRNAs have in proof-of-principle experiments been shown to lead to translation of protein, and this may occur naturally in tissues that have higher levels of circRNA expression, namely muscle and neuronal [27]. Several studies have identified small peptides formed from circRNAs that may have roles in cancer. For instance, an 87 amino acid long peptide from the circRNA formed from the lnc LINC-PINT suppresses glioblastoma proliferation, potentially through interactions with the Polymerase-associated factor 1 (PAF1) complex inhibiting oncogene transcriptional elongation [28]. Several other small peptides from circRNAs have been described with putative roles in cancer ranging from acting as a decoy for protein degradation mechanisms, increasing levels of the canonical protein [29], and activation of cancer signalling pathways, including Hippo and Wnt [30].

## 6. circRNA Facilitated Protein Interactions in Cancer

Other roles of circRNAs are mediated via protein interactions, including but not limited to sponging, forming scaffolds or decoys. circRNAs due to their covalently closed ring structure have the potential to form different RNA 3D structures compared to their linear transcripts, in turn potentially leading to completely different binding partners and novel interactions [31]. As a key role of circRNAs, it is therefore unsurprising that these interactions are found to be dysregulated in cancer.

Through these protein interactions, circRNAs are capable of widespread cellular dysregulation, for instance through epigenetic-mediated regulation of oncoproteins. As mentioned previously, the proto-oncoprotein FLI1 has been found overexpressed in breast and lung cancer, correlating with a poor prognosis. FLI1 overexpression has been linked to the function of a novel circRNA: circFECR1 [26]. circFECR1 binds to the promoter of DNA methyltransferase 1 (*DNMT1*) and recruits the Tet Methylcytosine Dioxygenase 1 (TET1) protein, TET1, through its demethylase activity, demethylates the CpG regions of the *DNMT1* promoter, resulting in decreased *DNMT1* transcription and therefore decreased protein levels, leading to demethylation of *FLI1* DNA, enhancing its expression. Through the binding and recruitment of TET1, circFECR1 has the potential to be a master regulator in breast cancer, aiding in oncogenesis.

A further key example of circRNA-protein interactions contributing towards oncogenesis is circDNMT1 expression in breast cancer. Cells with high levels of circDNMT1 have a higher senescence induction tolerance, allowing further replicative cycles and therefore promoting tumuorigenesis. Mechanistically, circDNMT1 binds to the protein Auf1. circDNMT1 through its binding to Auf1 leads to its nuclear translocation, preventing its usual cytoplasmic binding to *DNMT1* mRNA, thereby leading to increased DNMT1 stability and therefore increased translation of DNMT1. Increased levels of DNMT1 enhance *p53* promoter methylation in turn decreasing *p53* transcription and aiding tumour proliferation and survival [31].

circRNAs can conversely protect against and negatively regulate oncogenesis through specific protein interactions. circZKSCAN1 levels are reduced in hepatocellular carcinoma (HCC) through the activity of QKI5 and this reduction correlates with poorer survival rates. circZKSCAN1 binds to the protein FMRP acting as a competitive inhibitor, which prevents FMRP activating the downstream target CCAR1 which in turn prevents CCAR1 mediated activation of Wnt/β-catenin [32]. Furthermore, overexpression of circZKSCAN1 can lead to the inhibition of in vivo HCC growth in mouse xenograft models, highlighting the potential therapeutic value.

## 7. Fusion circRNAs in Cancer

The role of chromosomal translocations leading to the production of fusion gene products is well-known phenomenon in cancer, with examples including BCR-ABL and PML-RARA [33] with the resulting fusion protein contributing to or even driving oncogenesis. Recently evidence has emerged of chromosomal translocations leading to the formation of novel fusion circRNAs, which can drive oncogenesis. Screenings of clinical samples with known chromosomal translocations, including PML-RARα and MLL-AF9, identified several new fusion circRNAs. Interestingly, the fusion circRNAs formed showed variation from patient to patient, even if the patients had the same chromosomal translocation [34]. Furthermore, analysis of several fusion circRNAs found active roles in tumuorigenesis, rather than by-products of the initial translocation. These fusion circRNAs were artificially expressed in non-chromosomal translocated cells, allowing any changes in cell phenotype to be assigned to the circRNA, independent of the fusion protein or linear transcript. Cells had increased resistance to contact inhibition and showed increased proliferation upon activation of signalling pathways. Notably, targeted knockdown of specific fusion circRNAs expressed endogenously induced apoptosis, implicating these fusion circRNAs in cellular survival and as a potential cancer therapy target [34,35]. Furthermore, research has shown that the introduction of chromosomal translocations via CRISPR-Cas9 systems also leads to the formation of novel fusion circRNAs, implying circRNAs are likely produced from translocations events more readily than previously thought, with several different circRNAs produced from each translocation event, although functionality was not determined [36].

## 8. Exosomal circRNAs in Cancer

Exosomes are extracellular vesicles averaging around 40–160 nM in diameter, surrounded by a lipid bilayer. Discovered in the 1980s, their original role was thought to be restricted to cellular waste disposal. Since then, however, they are thought to function as key cellular messengers, transporting molecules including proteins, mRNA and metabolites all around the body [37]. As part of a cellular process, they have been found to be dysregulated during oncogenesis, aiding in communication between cancer cells and contributing to the tumour microenvironment. These exosomes have been designated tumour derived exosomes (TEXs) and deliver a range of factors, aiding growth, metastasis and other pro-oncogenic functions to cells and metastatic sites [38].

circRNAs have the potential to be key components of exosomes due to their inherent stability and multiple regulatory roles, with initial studies identifying the contents of tumour cell exosomes to vary from those of a healthy individual. Of particular note is the ratio of circRNAs to their linear counterparts in the tumour cell exosomes, which was up to six-fold higher, suggesting higher levels of incorporation against the natural cellular circBiome and therefore specific selection [39]. Furthermore, the exosome circBiome contents varies from the parent cells, once again suggesting specific incorporation of select circRNAs, over just random incorporation from the general population. However, it must be noted that circRNA enrichment in the exosomes could be due to their inherent stability and resistance to exonucleases, rendering them more resistant to degradation [40]. Finally, although there is potential for these exosomal circRNAs to simply be artifacts from an RNA disposal mechanism, numerous studies have found that RNAs packaged into exosomes have functionality in recipient cells. In addition, specific enrichment patterns suggest a form of selection, with similar studies in lncRNAs showing low abundance lncRNAs are highly enriched in exosomes, compared to higher abundance lncRNAs [41,42]. If exosomal circRNAs are just a mechanism of removal, it is likely the levels of RNAs within exosomes would reflect levels within the cell due to non-specific removal. However, current data does not suggest this.

Already there is functional analysis of specific exosomal enriched circRNAs in cancer, one study identified circPSMA1 as a circRNA enriched in cancer cell derived exosomes. Functional analysis showed this circRNA acted as a miRNA sponge, targeting the tumour suppressive miR-637, in turn leading to increase in Akt1 expression, and increases in β-catenin and cyclin D1 [43]. Due to circPSMA1 localisation to the exosomes, this pro-oncogenic mechanism was not just limited to the immediate area, with cells exposed to these circPSMA1 exosomes showing increased migration and proliferation in a dose-dependent manner.

circRNAs in exosomes can also aid in oncogenesis through mechanisms outside the primary hallmarks of cancer, with roles identified in the emerging hallmarks, namely glucose metabolism. Cancer cells have increased metabolic activity, and advanced cancer is often associated with cachexia. It is theorised this state can be due to increased levels of brown adipose tissues (BATs) formed from white adipose tissues (WAT) [44] which leads to increased energy expenditure. One study found gastric cancer patients had increased levels of BAT and proposed exosomal circRNAs could contribute, inducting BAT formation at sites far away from the primary tumour. Plasma exosomes had increased levels of ciRS-133. Moreover, levels of ciRS-133 correlated with BAT levels. The exosomes allowed the delivery of circRS-133 to the adipose sites, followed by increased sponging of miR-133. This in turn, led to the increased expression of PRDM16, a transcription factor, and increased maturation rates in adipocyte precursors [45].

Due to the ability of exosomes to migrate and play key roles as messengers, exosomes can particularly contribute to metastasis. Thereby, exosomal circRNAs also have the potential to aid in the formation of metastatic tumours, with the original primary tumour secreting pro-oncogenic circRNAs into exosomes to help prime a pre-metastatic niche in secondary locations around the body. A clinical example of these exosomal circRNAs playing a role in promoting metastasis would be circIARS. circIARS was found to be upregulated in pancreatic cancer patients’ tissues and furthermore enriched in exosomes found in the blood plasma. Higher levels of circIARS correlated with a poorer prognosis in patient survival and increased chance of metastasis from the primary tumour. Further experiments found exosomal circIARS was taken up by HUVEC cells at secondary sites, which led to increases in the permeability of the endothelial cells, aiding in the formation of metastatic sites. circIARS, once at the secondary sites, binds miR-122, in turn leading to increased levels of RhoA, increased F-actin and decreased ZO-1 to aid in endothelial cell monolayer permeability, thereby aiding metastatic spread [46]. Further, in vivo mouse work further suggested elevated circIARS can promote metastasis of tumours.

## 9. circRNAs as Potential Biomarkers and Therapeutics

The potential of circRNAs as biomarkers and therapeutics has gained significant interest in recent years due to their stability and specificity to certain diseases. Due to their closed structure, circRNAs are resistant to many cellular RNA decay mechanisms. Many studies have identified circRNAs to be specifically dysregulated or enriched in specific cancers. For instance, circPDE8A was found to be upregulated in exosomes released from pancreatic cancer cells, and through sponging of miR-338, it leads to the activation of MACC/MET/ERK and AKT pathways promoting invasion [47]. Of particular note is that the authors found the circRNA to be correlated with the severity of disease and survival rates, allowing the potential for future screens to indicate prognosis through use of the circRNA as a biomarker.

This excretion of circRNAs into exosomes is of particular interest if these circRNAs are excreted into the serum, plasma and blood, allowing non-invasive detection. Studies have already identified that circRNAs can be highly expressed in the saliva and the blood, allowing relatively non-invasive collection. Moreover, coupled with specificity for specific diseases and high stability, circRNAs have the potential to be ideal clinical biomarkers [48,49]. circRNAs have even been found to be detectable in patient urine [50]. Additionally, as previously discussed, several primary tumours excrete circRNAs in exosomes, aiding the establishment of metastatic sites. Metastasis is estimated to be responsible for 90% of cancer fatalities, often only detected past the point of treatment [51]. Monitoring of exosomes for known metastatic-promoting circRNAs would potentially allow for the detection of metastatic tumours early on in disease, allowing treatment [52]. Moreover, further studies are needed to investigate the specificity of circRNA dysregulation throughout stages of tumour development. Dysregulated circRNAs have been found to differ between the primary tumour site and any secondary sites. For instance, studies have found differentially expressed circRNAs in ductal carcinoma in situ (DCIS), the initial tumour site, compared to invasive ductal carcinoma (IDC) where the cancer has infiltrated surrounding tissue [53]. Interestingly, several conserved dysregulated circRNAs were also found between the DCIS and IDC tissues, implying that, despite circRNA dysregulation being highly specialised, there is the potential for biomarkers using a select few circRNAs. Likewise, there is evidence that circRNAs may be more conserved between primary and secondary sites than mRNAs, although whether this is a result of a consistent expression pattern or the increased stability of circRNAs has not yet been elucidated [54].

Already, levels of specific circRNAs correlate with severity of the disease and even survival rates. Three circRNAs are derived from the *PTGR1* gene, with increased levels in all three correlating with a poorer prognosis in HCC patients [55]. While increased levels of another, circPRMT5, were also associated with a poorer survival rate in bladder cancer [56]. Interestingly, both circRNAs were found to be upregulated in the tumour exosomes, once again implying circRNAs are specifically dysregulated in disease states and selected for exosomal packaging. This excretion into exosomes from the original tumour site would aid non-invasive detection of tumours and could be used for quick and regular screenings.

Given circRNA specificity in established cancers and metastatic tumours, there is also the potential for circRNA biomarkers to be used in early detection during pre-malignant disease states. For instance, several circRNAs have been found to be dysregulated in colorectcal adenoma that could act as predictive biomarkers for transition to colorectal cancer (CRC) [57]. Additional studies have also found examples of dysregulated circRNAs in other pre-cancerous tissues such as oral leukoplakia [58]. However, the most comprehensive and current circRNA sequencing focuses on established cancerous tissues and more research is needed into pre-malignant detection.

circRNA expression patterns have also been shown to be modulated under chemotherapy, with numerous instances of differences between circRNAs in chemoresistant compared to chemosensitive cells, from lung, to leukaemia and pancreatic [59]. However, whether these changes in circRNA expression patterns are contributing to chemoresistance or are simply by-products of other cellular changes requires further investigation. Nonetheless, initial studies suggest that at least some of these dysregulated circRNA actively promote chemoresistance. For instance, in cisplatin resistance cells, circRNA sponging of miR-296 leads to increased STAT3 levels, which are associated with increased resistance [60]. Thus, through monitoring of circRNAs, there is the potential for identifying chemoresistant cancer cells and tailoring treatment accordingly. Alternatively, circRNAs have also been identified that can reverse drug resistance. One example is circMTO1, which reverses drug resistance to monastrol in breast cancer. Monastrol inhibits the function of the protein Eg5, also known as kinesin-5, and prevents its activity within spindle formation. This leads to spindle formation arrest and increased apoptosis. However, resistance to monastrol can occur through mutations in Eg5 preventing drug binding. Research has found overexpression of circMTO1 can counteract this drug resistance, through binding to TNF receptor-associated factor 4 (TRAF4), in turn preventing TRAF4-mediated promotion of Eg5 transcription, leading to reduced Eg5 levels [61], although whether this mechanism is functionally in vivo is not elucidated.

Additionally, utilising circRNAs ability to sponge miRNA as a means of downregulating signalling pathways involved in cancer has been investigated as a novel therapeutic approach. miRNA sponges can be expressed as miRNA sponge cassettes that are formed of tandem repeats of identical miRNA targeting sites. These cassettes sequester endogenous cellular miRNAs, impairing the binding of the miRNA to the natural target, thus inhibiting the miRNAs post transcriptional regulation activity [62]. Advances in miRNA sponging lead to the development of artificial circRNA sponges. circmiR was expressed endogenously in a mammalian model system designed with targets against known cardiac pro-hypertrophic miR-132 and miR-212. circmiR showed successful antagonism of target miRNAs and attenuated pathological hypertrophy in a mouse model [63].

A final aspect of circRNAs therapeutic approaches is the potential to act as vectors for protein expression to aid in the treatment of disease. Currently, the majority of research is focused on the use of mRNA. However, some of the major hurdles to using mRNA include its natural instability and ability to trigger an immune response [64]. Evidence suggests that circRNAs can evade RNA sensing mechanisms within the cell, with the introduction of exogenous circRNAs leading to significantly less activation of RIG-I and Toll-like receptors compared to linear RNA [65]. Moreover, due to their stability, exogenous circRNAs engineered to produce proteins were found to have a protein-producing half-life of 80–116 h dependent on cell line, compared to 45–50 h using exogenous linear vectors. This increased half-life not only compensated for the decreased efficiency of translation associated with circularisation, but led to increased protein yields compared to linear vectors. However, just as the overall area of protein expression therapy requires further investigation, so does the use of circRNAs, with particular problems in guaranteeing circularisation efficiency [66].

## 10. Conclusions

The study of circRNA dysregulation in cancer is an emerging field that offers exciting new avenues of research. The multitude of functions that circRNA perform within cellular biology has shown the breadth of dysregulation that can occur in many cancers and other disease states. Evidently, these functions are complex and intertwined and have a profound effect on signalling pathways, transcription and translation when dysregulated. Many questions remain concerning circRNAs, including the causal factors of dysregulation and how big a role they may play in driving oncogenesis. The potential to utilise the intrinsic stability and specificity of circRNAs for diagnostics and treatment in particular merits further investigation.
